# Cardiac Failure Forecasting Based on Clinical Data Using a Lightweight Machine Learning Metamodel

**DOI:** 10.3390/diagnostics13152540

**Published:** 2023-07-31

**Authors:** Istiak Mahmud, Md Mohsin Kabir, M. F. Mridha, Sultan Alfarhood, Mejdl Safran, Dunren Che

**Affiliations:** 1 Department of Electrical and Electronic Engineering, Ahsanullah University of Science and Technology, Dhaka 1208, Bangladesh; istiakmahmud.eee@gmail.com; 2 Department of Computer Science and Engineering, Bangladesh University of Business and Technology, Dhaka 1216, Bangladesh; mdmkabi@gmail.com; 3 Department of Computer Science, American International University-Bangladesh, Dhaka 1229, Bangladesh; 4 Department of Computer Science, College of Computer and Information Sciences, King Saud University, P.O. Box 51178, Riyadh 11543, Saudi Arabia; mejdl@ksu.edu.sa; 5 School of Computing, Southern Illinois University, Carbondale, IL 62901, USA; dche@cs.siu.edu

**Keywords:** cardiac failure, metamodel, forecasting, random forest classifier, decision tree, Gaussian Naive Bayes, machine learning, k-Nearest Neighbour

## Abstract

Accurate prediction of heart failure can help prevent life-threatening situations. Several factors contribute to the risk of heart failure, including underlying heart diseases such as coronary artery disease or heart attack, diabetes, hypertension, obesity, certain medications, and lifestyle habits such as smoking and excessive alcohol intake. Machine learning approaches to predict and detect heart disease hold significant potential for clinical utility but face several challenges in their development and implementation. This research proposes a machine learning metamodel for predicting a patient’s heart failure based on clinical test data. The proposed metamodel was developed based on Random Forest Classifier, Gaussian Naive Bayes, Decision Tree models, and k-Nearest Neighbor as the final estimator. The metamodel is trained and tested utilizing a combined dataset comprising five well-known heart datasets (Statlog Heart, Cleveland, Hungarian, Switzerland, and Long Beach), all sharing 11 standard features. The study shows that the proposed metamodel can predict heart failure more accurately than other machine learning models, with an accuracy of 87%.

## 1. Introduction

Heart failure is a complex and potentially life-threatening condition that significantly burdens healthcare systems worldwide. It is a pathophysiologic condition in which the heart’s inability to pump blood at a rate sufficient to meet the needs of the body’s metabolizing tissues results from faulty cardiac function [1,2]. It includes a number of heart-related illnesses, such as coronary artery disease, heart attacks, heart failure, arrhythmias, and several other cardiovascular ailments. Heart disease is a leading cause of death globally [3], accounting for many premature deaths and posing a significant burden on healthcare systems. Heart disease is a common and significant health issue in many parts of the world [4]. The American Heart Association says that heart failure is projected to increase dramatically [5]. Accurate prediction of heart failure can play a vital role in early detection and prevention of adverse outcomes, ultimately leading to improved patient outcomes and reduced healthcare costs.

Timely and accurate detection of heart failure is crucial for effective management and treatment [6]. Detecting heart failure early allows for prompt intervention and the implementation of appropriate medical strategies, which can help slow the progression of the disease, alleviate symptoms, and improve the patient’s quality of life. Early detection can also reduce the risk of complications and hospitalizations associated with advanced stages of heart failure. From 1989 [7] to now, there have been many approaches to finding the best methods for cardiac failure prediction. In 2017, Simge et al. [8] used Matlab and WEKA to find the best way to detect heart failure disease and obtained a good accuracy of 67.7% for the ensemble subspace discriminant algorithm and the decision tree algorithm. Then, in 2018, Ali et al. [9] utilized the Claveland dataset [10] for their studies and obtained 84% accuracy for the Naive Bayes algorithm. Further, in 2019, Saba et al. [11] performed prediction for heart diseases and obtained 84.85% accuracy for the logistic regression (SVM) technique. However, most of them used the same dataset from the UCI repository [12], which contains 300 records. This is a rather limited amount of data for machine learning training.

Machine learning techniques have drawn a lot of attention in the medical field lately because of their potential to help with the detection and prediction of cardiac disease [13]. Large volumes of clinical data may be analysed by machine learning algorithms to find links and patterns that are not immediately obvious to human practitioners [14]. These algorithms can harness the power of computer models to make accurate predictions and provide valuable insights into disease risk assessment. However, the development and implementation of machine learning models for heart failure prediction face several challenges [15]. The complexity of the cardiovascular system and the multifactorial nature of heart failure necessitate integrating diverse data sources, including clinical test data, medical imaging, and patient demographics. Data quality, feature selection, and model performance issues must be addressed to ensure reliable and clinically relevant predictions.

This research addresses these challenges by proposing a machine learning metamodel for predicting heart failure based on clinical test data. The metamodel incorporates several established machine learning algorithms, namely the Gaussian Naive Bayes (GNB), Random Forest Classifier (RFC), Decision Tree models (DT), and k-Nearest Neighbor (KNN), to leverage their individual strengths in classification tasks. Combining these models into a metamodel aims to enhance predictive accuracy and robustness while reducing potential biases associated with individual algorithms. To evaluate the performance of the proposed metamodel, a combined dataset comprising five well-known heart datasets, including the Statlog Heart, Cleveland, Hungarian, Switzerland, and Long Beach datasets, is utilized. These datasets share 11 standard features such as age, chest pain type, sex, resting BP, fasting BS, cholesterol, resting ECG, exercise angina, maxHR, oldpeak, and ST-slope, widely used in previous heart disease prediction studies. By leveraging a diverse set of data sources, the metamodel aims to represent the underlying factors contributing to heart failure comprehensively. The overall contribution of this paper includes the following:

- Integrate common machine learning algorithms such as Random Forest Classifier, Gaussian Naive Bayes, decision tree models, and k-Nearest Neighbor into a metamodel framework, leveraging their strengths to improve predictive accuracy and model robustness.
- We have used a combined dataset from five different and well-known cardiac datasets, including Statlog Heart, Cleveland, Hungary, Switzerland, and Long Beach, to ensure a comprehensive representation of patient characteristics, clinical features, and risk factors and improve the metamodel’s generalizability and applicability.
- The performance and evaluation metrics of the proposed metamodel have been compared with other state-of-the-art machine learning models.

The structure of the paper is as follows: In Section 2, a summary of the related works is provided. Section 3 outlines the methodology used. The results are presented in Section 4. Section 5 focuses on the discussion. Finally, Section 6 serves as the conclusion of the paper.

## 2. Related Works

Heart failure forecasting has garnered significant attention recently due to its potential to enhance patient care and improve healthcare resource allocation. Numerous studies have explored the application of machine learning and deep learning techniques in predicting the onset and progression of heart failure. These methods leverage the abundance of clinical and physiological data available, aiming to provide early and accurate prognostic insights for clinicians and patients. In this section, we review the literature on heart failure forecasting, focusing on the various machine learning and deep learning approaches employed, the datasets utilized, and the reported performance metrics.

Liang et al. [16] proposed a novel deep learning model called tBNA-PR to accurately predict heart failure and identify sub-phenotypes using temporal electronic health records (tEHRs) data. The model effectively captures the complexity and heterogeneity of the data to obtain informative patient representations. The study demonstrates the effectiveness of tBNA-PR on a real-world dataset, achieving prediction accuracy of 0.78, F1-Score of 0.7671, and AUC of 0.7198, outperforming existing benchmarks. The analysis identifies three distinct sub-phenotypes of heart failure patients based on clustering and subgroup analysis, revealing specific characteristics and significant features associated with each sub-phenotype. The findings have practical implications for clinical decision support, but the study acknowledges limitations related to data completeness, disease specificity, generalizability, interpretability, and the need for further research.

In a study by Robert et al. [7], a novel algorithm was proposed for diagnosing coronary artery disease, employing a probability-based approach. This algorithm’s reliability and clinical utility were tested across three patient test groups. 303 consecutive patients who were sent for coronary angiography at the Cleveland Clinic between May 1981 and September 1984 served as the reference group for the model’s development. The study’s findings showed that when applied to individuals with chest pain syndromes and intermediate disease prevalence, discriminant functions used to determine coronary disease probabilities produced accurate and clinically helpful results. In another study by Simge et al. [8], a comparison was made between two prominent machine learning platforms using the same dataset. The researchers conducted experiments to classify heart disease using six distinct algorithms: Quadratic SVM, Linear SVM, Cubic SVM, Decision Tree, Medium Gaussian SVM, and Ensemble Subspace Discriminant. These experiments were carried out in both the Matlab environment and WEKA. The dataset utilized in this study was acquired from the machine learning repository of UCI [12]. The highest accuracy achieved was 67.7% using the Ensemble Subspace Discriminant algorithm in Matlab, while the Decision Tree algorithm in the WEKA platform also yielded an accuracy of 67.7%.

Li et al. [17] introduce a deep learning-based automatic system for diagnosing heart failure by tackling the issue of imbalanced data in chest X-ray (CXR) images. The approach combines under-sampling and instance selection techniques to maintain the integrity of data distribution and presents a comprehensive multi-level classification method to diagnose specific heart failure causes. Experimental results demonstrate that the proposed approach outperforms traditional under-sampling methods, achieving an accuracy of 84.44% in multi-class classification tasks. Rao et al. [18] presented a deep-learning framework for predicting heart failure incidence using electronic health records. The authors developed a novel Transformer-based risk model incorporating patient diagnoses, medications, age, and calendar year. The model achieved high predictive performance, outperforming existing deep learning models. Ablation analysis revealed the importance of medications and calendar year in predicting HF risk. Contribution analyses identified both established risk factors and new associations, providing insights for data-driven risk factor identification. The study highlights the potential of the deep learning model to inform preventive care and identify new hypotheses for further research and drug repurposing studies in HF prediction and other complex conditions.

In a related study conducted by Ali et al. [9], the Cleveland dataset was employed for analysis, and a feature selection process was carried out to train three distinct classifiers, namely Support Vector Machine (SVM), Naïve Bayes, and K-Nearest Neighbors, utilizing a 10-fold cross-validation technique. Their findings revealed that the Naïve Bayes classifier exhibited superior performance on this dataset and the selected features, surpassing or equaling the performance of SVM and KNN across all four evaluation parameters. Notably, it achieved an accuracy rate of 84%. Saba et al. conducted a study [11] that explores the prediction of heart disease using data science methodologies. Their research focuses on employing feature selection techniques and algorithms to improve the accuracy of heart disease prediction. Multiple heart disease datasets were utilized for experimentation and analysis purposes. The authors employed various feature selection techniques, including Logistic Regression, Decision Tree, Random Forest, Nave Bayes, and Logistic Regression SVM, using Rapid Miner as the tool. Notably, the highest achieved accuracy of 84.85% was obtained using the UCI dataset [12] in combination with the Logistic Regression (SVM) technique.

Earlier research on predicting heart failure has mostly relied on two widely recognized datasets, namely the UCI repository and the Cleveland dataset, as indicated in Table 1. However, these datasets suffer from limitations in terms of the number of records available for machine learning training purposes. Additionally, prior investigations primarily employed basic machine learning models for detection or forecasting tasks. To address these limitations, we conducted our research using a comprehensive dataset comprising 918 records and introduced a novel metamodel for predicting cardiac failure in patients. Our metamodel represents a fusion of four distinct machine-learning models, allowing for enhanced accuracy and robustness in forecasting outcomes.

## 3. Methodology and Materials

**Figure 1:**
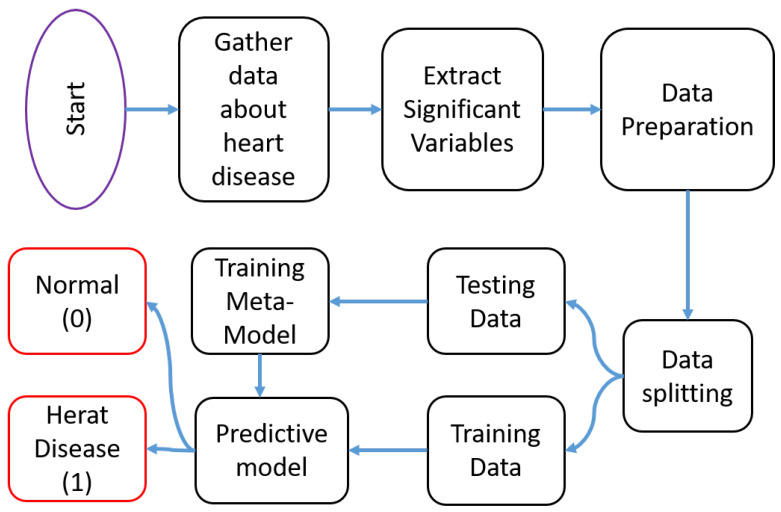
The flow diagram of the proposed system.

The methodology employed in this study aims to develop and evaluate a machine learning metamodel for predicting heart failure based on clinical test data. The flow of the proposed framework is depicted in Figure 1. The first step involves data collection to create the dataset. Next, significant variables are extracted, and the data are prepared accordingly. Subsequently, the dataset is divided into training and testing sets. The training data are then utilized to train the proposed metamodel. Finally, the metamodel is generated and tested to obtain the output results.

### 3.1. Dataset and Attributes

**Figure 2:**
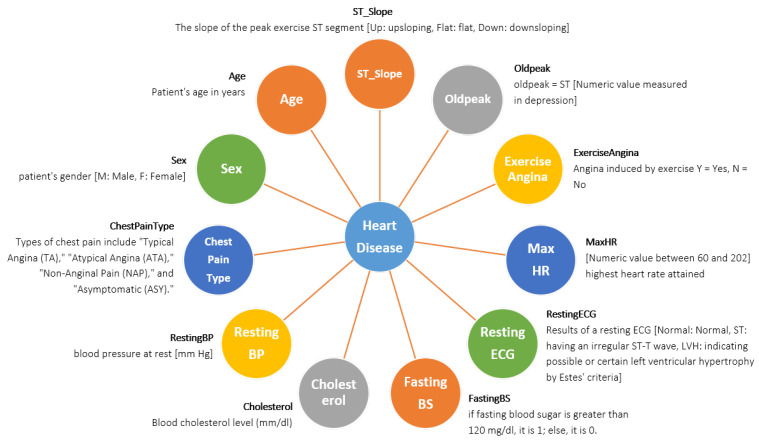
The details of the features used in this system model are illustrated in the diagram.

The creation of this dataset involved the integration of multiple existing datasets that had not been previously combined. Currently, this dataset stands as the most extensive resource available for heart disease research, as it merges five distinct heart datasets (Statlog (Heart) Data Set: 270 records, Cleveland: 303 records, Hungarian: 294 records, Long Beach, VA: 200 records, Switzerland: 123 records) and shares 11 common features. The dataset is accessed from Kaggle named ‘Heart Failure Prediction Dataset’ [25]. The dataset contains 920 patient records, including 725 males and 195 females of different ages. Where 267 males are normal, and 458 males have heart disease, 145 females are normal, and 50 females have heart disease. The comprehensive depiction of every attribute, along with the corresponding count of values for each attribute, can be observed in the provided Figure 2.

### 3.2. Data Preprocessing

**Figure 3:**
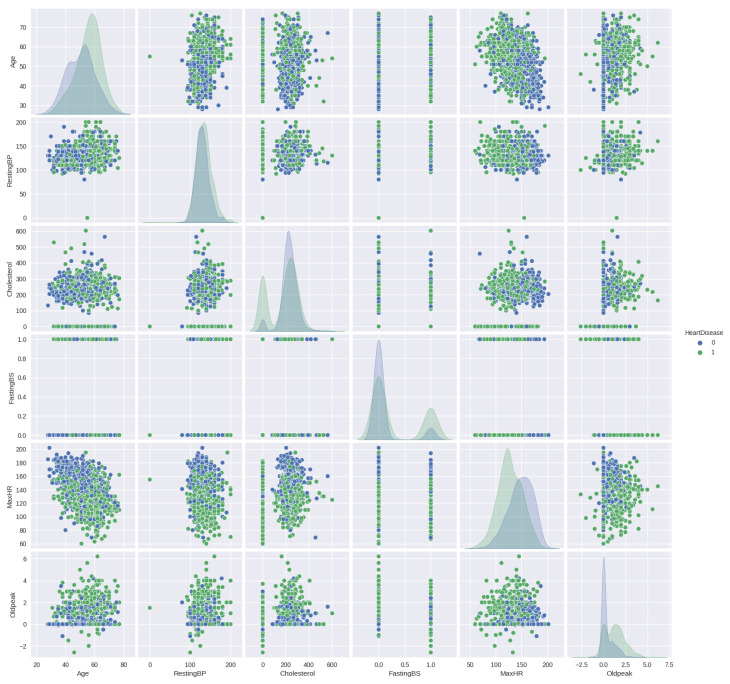
Pairplot between different features with respect to the heart disease data. The plot displays the pairs of variables in the dataset that exhibit the highest correlation. It indicates that accurately predicting the final classification using a two-parameter set is particularly challenging. In this context, the value of 0 represents a normal patient, while the value of 1 corresponds to a patient with heart disease.

Data preprocessing plays a crucial role in machine learning [26], and its importance cannot be overstated. To enable the machine to learn from the data and generate the suitable model, it is crucial to convert the categorical feature values into numerical representations through a process known as an encoding [27] method, which is utilised here [28]. The data collected in this stage is injected into the Google Colab platform in Python programming to acquire the desired output [29]. The dataset demonstrates that the independent variables have a significant impact on analyzing the relationship between them and the output variable. In this case, the output variable consists of only two options. Figure 3 displays the pairplot of the numerical features in relation to HeartDisease. Figure 3 highlights the pairs of variables that exhibit the strongest correlation in the dataset. From the plot, it becomes evident that predicting the final classification based on the two-parameter set is challenging.

### 3.3. Baseline Architectures

This section introduces the fundamental architectures employed in the metamodel, namely Random Forest, Naive Bayes, and Decision Tree.

#### 3.3.1. Random Forest

Random Forest is a technique for ensemble learning that combines multiple decision trees to make predictions collectively [30]. In Random Forest, each decision tree makes individual predictions, and the final prediction is obtained by aggregating the predictions of all the trees. Let us denote the Random Forest model as RF, the input features as *X*, and the target variable as *Y*. Assuming we have *N* decision trees in the forest, the prediction of RF can be represented as follows:

(1) $$RF\left(X\right)=mode(Tree_{1}\left(X\right),Tree_{2}\left(X\right),...,Tree_{N}\left(X\right))$$

where $Tree_{i}\left(X\right)$ represents the prediction of the *i*-th decision tree. In a classification task, mode() returns the most frequent class label among the predictions of all trees. In a regression task, mode() can be replaced by averaging the predictions. Every decision tree is created using a bootstrapped subset of the training data, and each node’s predictions are based on a random selection of characteristics. The aggregation of predictions allows the Random Forest model to reduce overfitting and improve generalization performance.

#### 3.3.2. Naive Bayes Classifiers

Naive Bayes is a probabilistic classifier that operates under the assumption of feature independence given the class label [31]. Let us denote the Naive Bayes classifier as NB, the input features as *X*, and the class label as *Y*. The classification task aims to predict the probability of a class given the input features. Using Bayes’ theorem, this probability can be calculated as follows:

(2) P(Y|X)=P(X|Y)*P(Y)/P(X)

In Gaussian Naive Bayes, the assumption is that the continuous features *X* follow a Gaussian distribution. The probability P(X|Y) is estimated by fitting a Gaussian distribution for each class *Y*, with mean $\mu_{Y}$ and standard deviation $\sigma_{Y}$. The prior probability P(Y) is estimated based on the frequency of each class in the training data. The probability P(X) is a normalization constant that can be ignored in the classification decision. To predict the class label for a new instance *X*, the classifier selects the class *Y* with the highest posterior probability P(Y|X) using the maximum a posteriori (MAP) estimation:

(3) NB(X)=argmaxP(Y|X)=argmaxP(X|Y)∗P(Y)

where argmax() returns the class label that maximizes the expression. Equation (4) displays the probability determined by GNB.

(4) $$\begin{array}{cc} P(x_{i}|y) & =\frac{1}{\sqrt{(}2\pi \sigma_{y}^{2})}\exp\left(-\frac{{\left(x_{i}-\mu_{y}\right)}^{2}}{2\sigma_{y}^{2}}\right)\\ \mathrm{Here},\;P(x_{i}|y)\; & =\mathrm{Probability}\;\mathrm{of}\;x_{i}\;\mathrm{occurring}\;\mathrm{given}\\ \; & \mathrm{evidence}\;y\;\mathrm{has}\;\mathrm{already}\;\mathrm{occurred}\;\\ \sigma & =\mathrm{Standard}\;\mathrm{Deviation}\\ \mu & =\mathrm{Mean} \end{array}$$

#### 3.3.3. Decision Tree

A decision tree is a hierarchical structure that uses a series of feature tests to make predictions [32]. Let us denote the decision tree as DT, the input features as *X*, and the target variable as *Y*. The decision tree recursively splits the dataset based on feature tests, aiming to maximize the separation of classes or minimize impurity. The prediction of the decision tree can be represented as follows:

(5) $$DT\left(X\right)=\sum_{i=1}^{L}y_{i}\cdot I\left(X\in R_{i}\right)$$

Here, *L* represents the number of leaf nodes in the decision tree, $y_{i}$ represents the class label assigned to the *i*-th leaf node, and $R_{i}$ represents the region or subset of instances assigned to the *i*-th leaf node based on the feature tests. $I(X\in R_{i})$ is an indicator function that returns 1 if the input instance *X* belongs to the region $R_{i}$ and 0 otherwise. The decision tree traverses from the root to a leaf node based on the feature tests and assigns the corresponding class label $y_{i}$ for the leaf node in which the instance falls.

### 3.4. Proposed Metamodel Architecture

The main dataset consists of 920 data points, equating to 920 rows and 12 columns representing various variables such as Age, ChestPainType, Sex, RestingBP, FastingBS, RestingECG, Cholesterol, MaxHR, ST-Slope, Oldpeak, ExerciseAngina, and HeartDisease. In the case of the metamodel, the primary dataset is divided into two parts: one for training and the other for testing. The training portion comprises 736 rows, while the testing portion consists of 184 rows. Hence, the training data have a structure of (736, 12), and the testing data have a structure of (184, 12).

To prevent overfitting in the stacking method, K-Fold Cross-validation was employed [33]. In this case, the value of K was set to 4, resulting in subsets of 184 rows each. During each iteration of the cross-validation process, four subsets were utilized for training and one subset for testing, with a unique test set assigned for each iteration. Following the K-Fold Cross-validation, three new outcomes were obtained, namely the predicted data from the Random Forest Classifier, Gaussian Naive Bayes, and Decision Tree models. Subsequently, the ‘HeartDisease’ column from the primary training dataset was included to make predictions for the metamodel, which in this case is KNN. The KNN algorithm can be expressed as follows:

(6) $$\begin{array}{cc} d(x,y) & =\sqrt{(}\sum_{i=1}^{n}{\left(x_{i}-y_{i}\right)}^{2})\\ \mathrm{Here},\;n & =\mathrm{no}\;\mathrm{of}\;\mathrm{dimensions}\;\left(n=6\right)\\ x & =\mathrm{data}\;\mathrm{point}\;\mathrm{from}\;\mathrm{dataset}\\ y & =\mathrm{new}\;\mathrm{data}\;\mathrm{point}\;(\mathrm{to}\;\mathrm{be}\;\mathrm{predicted}) \end{array}$$

**Figure 4:**
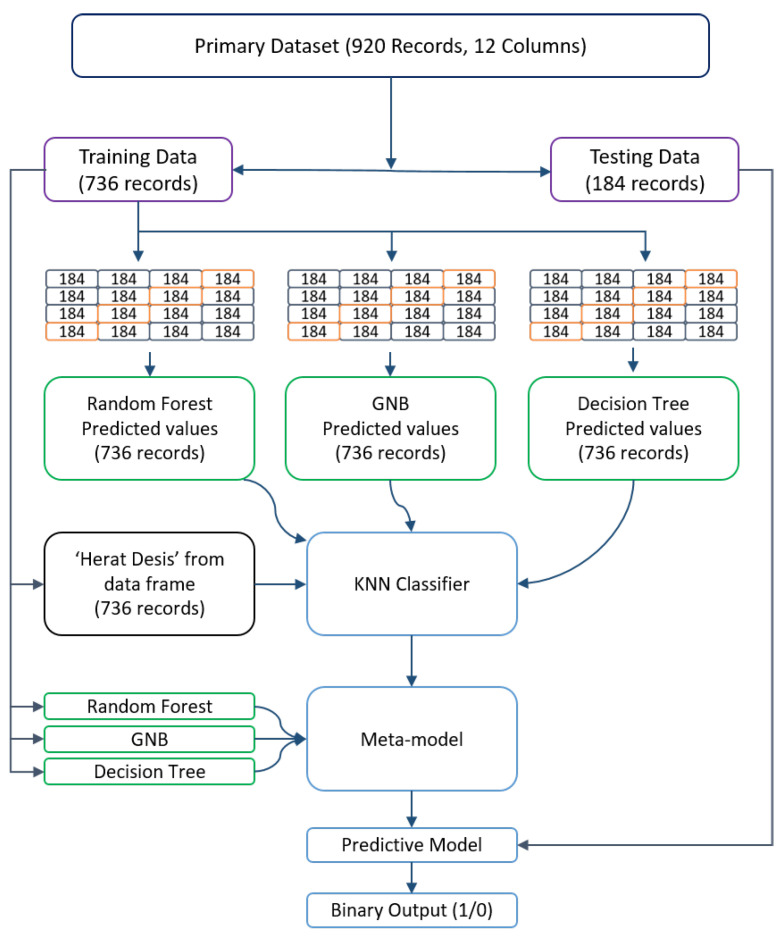
The meta-general model’s mechanism is shown in the figure. Distinctly colored boxes symbolize the specific operations.

The Random Forest Classifier, Gaussian Naive Bayes, and Decision Tree models are employed as estimators, while K-Nearest Neighbors serve as the final estimator. The resulting data structure becomes (736, 4), with four columns representing the predicted results from the Random Forest Classifier model, Gaussian Naive Bayes model, Decision Tree model, and the ‘HeartDisease’ column of the primary training dataset. These four columns are then used to prepare the metamodel. To obtain the base models, the primary training dataset needs to be trained using the three fundamental models: RFC, GNB and DT. This process allows us to derive the model for predicting heart failure. Finally, the primary test data are passed through the final model to validate and assess the data. The overall model structure is depicted in Figure 4.

## 4. Experimental Results

In this section, we will discuss the experimental setup, evaluation metrics, statistical data analysis, the performance of the proposed metamodel, and a comparison of the metamodel with other state-of-the-art approaches.

### 4.1. Experimental Setup

Python was chosen as the programming language for implementing the metamodel, and the implementation process involved utilizing the sci-kit learn library. The metamodel was trained in the Google Colab environment.

### 4.2. Evaluation Metrics

To determine the best-performing algorithm, a number of detection algorithms were performed to the dataset and their results were compared for accuracy and other statistical factors. The algorithms used were a decision tree, bagging classifier, LGBM, Ridge classifier, SVR, SGDC, KNN, GPC, and a blended metamodel. Based on the metrics used to evaluate their performance, these algorithms were compared. This subsection gives a brief description of various performance matrices.

#### 4.2.1. Accuracy

The percentage of accurate predictions to all predictions is used to produce the classification accuracy rating, often known as the accuracy score. Equation (7) defines accuracy (*A*).

(7) $$\begin{array}{cc} A & =\frac{True\;Positive+True\;Negative}{Total\;Number\;of\;Predictions} \end{array}$$

#### 4.2.2. Precision

The proportion of true positive results divided by the total quantity of positive outcomes, including misdiagnosed ones, is used to calculate precision (*P*). Calculating *P* involves using Equation (8):

(8) $$\begin{array}{cc} P & =\frac{True\;Positive}{True\;Positive+False\;Positive} \end{array}$$

#### 4.2.3. Recall

The recall is calculated as the ratio of real positive samples that should have been identified to genuine positive findings. Equation (9) is used to calculate the recall:

(9) $$\begin{array}{cc} R & =\frac{True\;Positive}{True\;Positive+False\;Negative} \end{array}$$

#### 4.2.4. F1-Score

The F1 score determines the accuracy of the model in each class. When the dataset is unbalanced, the F1-score metric is often applied. Here, it illustrates the effectiveness of the suggested strategy using the F1 score as an assessment indicator [34]. Equation (10) is used to obatin the F1-score.

(10) $$\begin{array}{cc} F1 & =2\times \frac{precision\times recall}{precision+recall} \end{array}$$

### 4.3. Statistical Data Analysis

**Figure 5:**
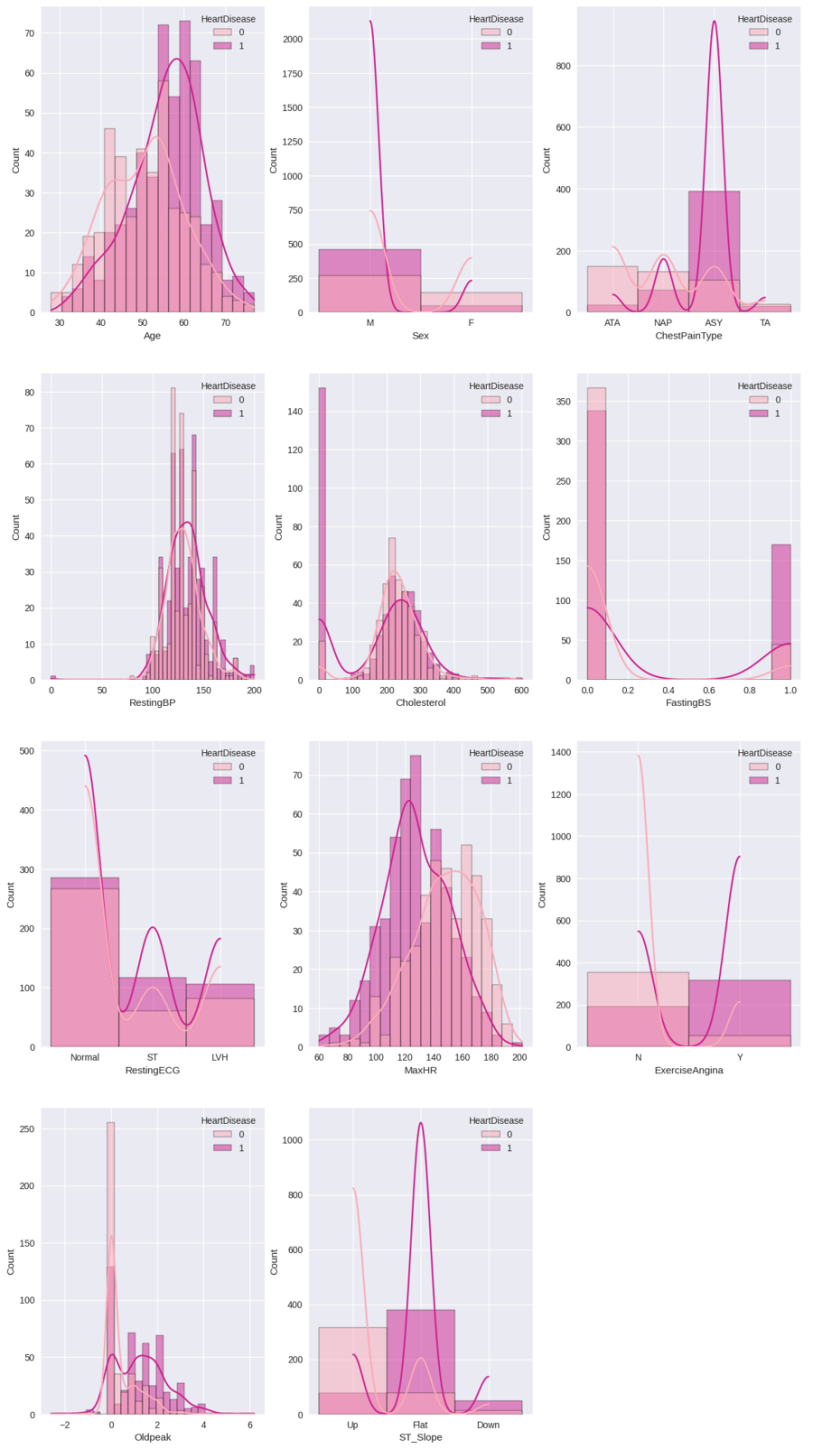
Relationship between different features with respect to heart disease results.

Figure 5 illustrates the relationship of heart disease with all other features used in the dataset. Here, it is clear that people aged 50–70 are more affected by heart disease than others. The data highlights that men are more likely to be affected by the disease than women. More people had symptoms of chest pain that were type ‘ASY’. The cholesterol data for heart disease patients was approximately between 180–300. For the majority of individuals with heart disease, the slope was flat, with a maximum heart rate of 100–130.

**Figure 6:**
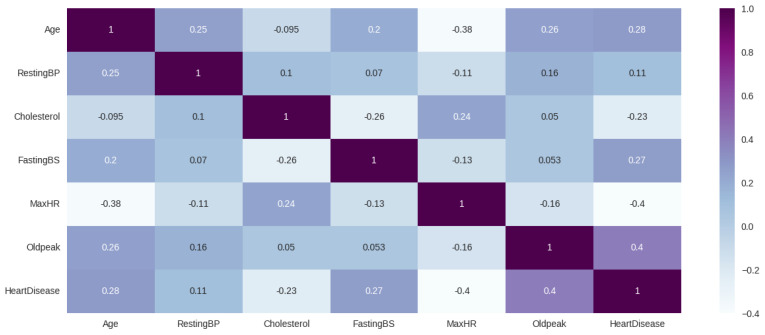
Heatmap represents the correlation matrix between different features.

Figure 6 shows the pairwise correlation between the used features of the dataset. Here, each cell in the matrix represents the correlation coefficient, which indicates the strength and direction of the linear relationship between two features. From the figure, it is evident that age, oldpeak, resting BP, and fasting BP are all positively correlated with heart disease, while cholesterol and maxHR are negatively correlated.

**Figure 7:**
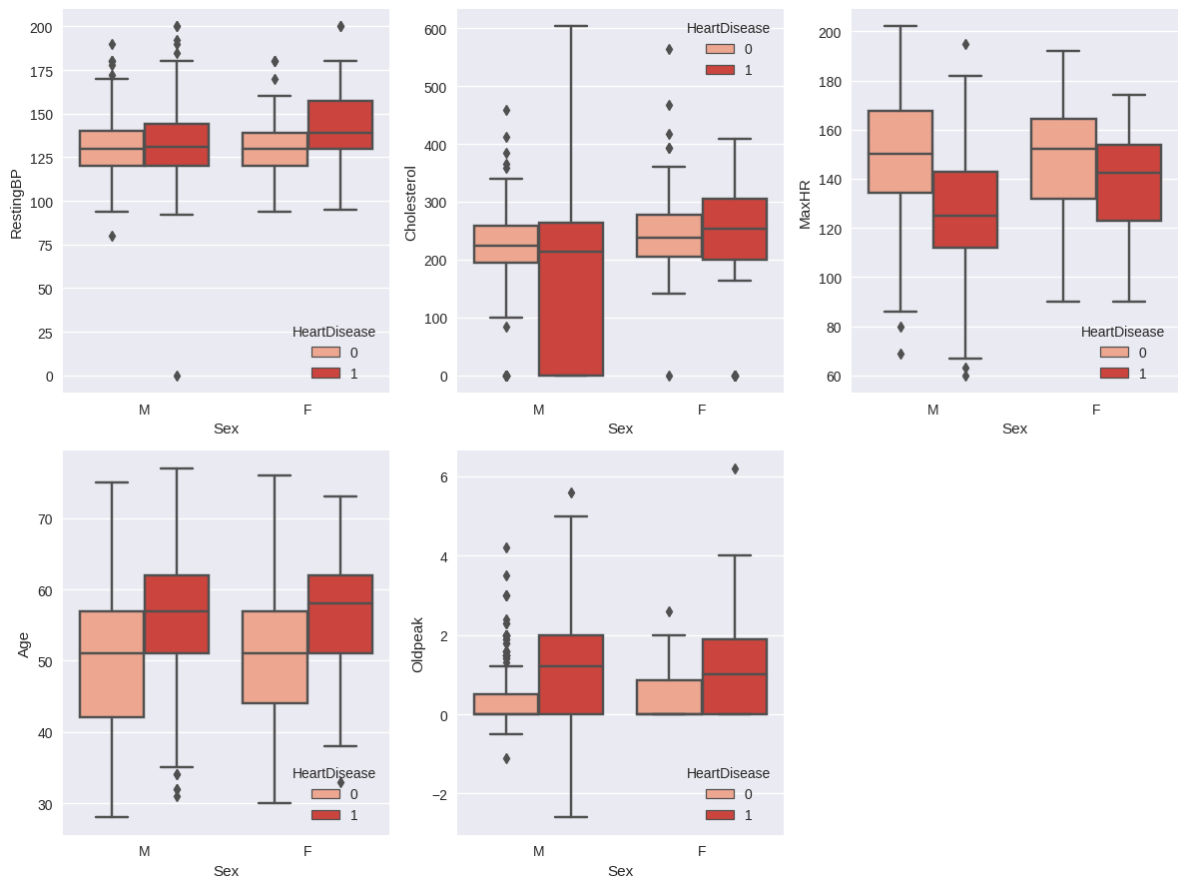
Relationship between heart disease by gender and other features.

The relationship between heart disease by gender and other features is shown in Figure 7. The resting blood pressure is almost the same for the male patient with heart disease and the normal patient, whereas the female patient with heart disease has a resting blood pressure between 130–160 and the normal female patient has a resting blood pressure between 120–140. Male patients with heart disease have elevated cholesterol levels, which are considered normal in the range of 190–260. The maximum heart rate is normally decreased in the case of an abnormal heart condition in both genders.

### 4.4. Performance of the Metamodel

The metamodel we have suggested achieves an accuracy, recall, precision, and F1-score of 87% when applied to our processed dataset. We evaluated our proposed model against various machine learning models including DT, Bagging classifier, LGBM, Ridge classifier, SVR, SGDC, KNN, and GPC. The results, showcasing the accuracy, precision, and recall metrics, can be found in Table 2. Table 2 clearly demonstrates that the proposed metamodel outperforms all other base models in terms of accuracy, precision, recall, and F1-score.

**Figure 8:**
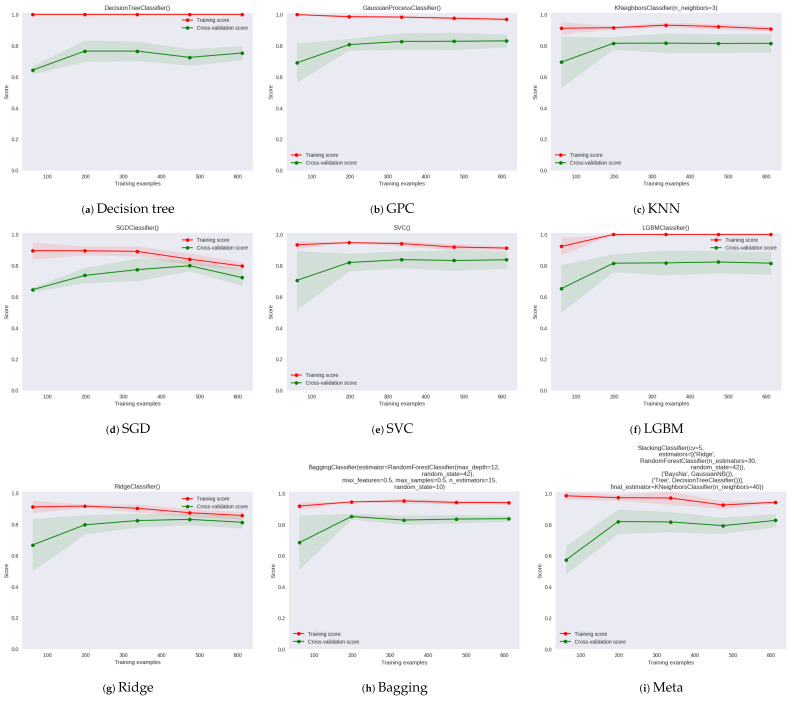
Learning plots for different machine learning models and proposed metamodel.

In Figure 8, the learning curve provides information about the performance and behavior of a model as the amount of training data increases. It illustrates the relationship between the training set size, the number of training iterations, and the model’s performance metrics, such as accuracy, error, or loss. In the plot, the y axis represents training and cross-validation scores, and the x axis represents the training examples for different machine learning models. The learning curve for GPC model in Figure 8 shows that it has the lower test variability and a low score up to 200 instances and the final F1 score is 0.61. The learning curves of Bagging, Ridge, and LGBM show high test variability and resulted in F1 scores of 0.86, 0.86, and 0.83, respectively. The proposed metamodel learning curve shows the highest test variability and a low score up to around 200 instances; however, after this level, the model converges on an F1 score of around 0.87.

Table 3 summarizes the parameters, parameter counts, dataset records, and model comparison for different studies in the field. The table includes multiple studies, each with its dataset and algorithm used. The parameters used in each study are listed, along with the corresponding count of parameters. The number of records or data points in the dataset is also mentioned. The algorithms employed in each study are indicated, along with their corresponding accuracy percentages. The table showcases a comparison of the proposed system with other studies. The proposed system utilizes the “fedesoriano” dataset [25] and employs a metamodel. It utilizes 11 parameters and consists of 918 records. The accuracy of the proposed system is reported as 87%, which is higher than the accuracy percentages achieved by other models in the table. This comparison demonstrates the superior performance of the proposed system when compared to earlier research and models.

## 5. Discussion

Developing a machine learning metamodel for cardiac failure forecasting based on clinical data represents a significant advancement in cardiovascular medicine. This discussion section will dive into the key findings of this research, discuss the implications for clinical practice, highlight the strengths and limitations of the metamodel, and suggest avenues for future research.

The evaluation of the proposed metamodel revealed its superior performance compared to other state-of-the-art machine learning models. With an accuracy of 87%, the metamodel showcased its potential for accurately predicting heart failure based on clinical test data. This high level of accuracy is promising, as it has the potential to aid healthcare professionals in identifying patients at risk of heart failure and implementing preventive measures in a timely manner. Early detection of heart failure is crucial for initiating appropriate interventions and personalized treatment plans, ultimately leading to improved patient outcomes.

One of the strengths of the metamodel lies in its incorporation of multiple machine learning algorithms, namely Random Forest Classifier, Gaussian Naive Bayes, decision tree models, and k-Nearest Neighbor. By blending these algorithms, the metamodel leverages their individual strengths, such as the ability of decision trees to capture complex interactions and the robustness of Random Forest Classifier in handling noisy data. This integration enhances the metamodel’s predictive accuracy and model robustness, making it a valuable tool for forecasting cardiac failure.

Utilizing a combined dataset from five well-known cardiac datasets, including Statlog Heart, Cleveland, Hungarian, Switzerland, and Long Beach, ensures a comprehensive representation of patient characteristics, clinical features, and risk factors. This approach enhances the generalizability and applicability of the metamodel, as it captures a diverse range of patient profiles and healthcare settings. Including 11 standard features from these datasets provides a solid foundation for predicting heart failure, but future studies can explore the integration of additional clinical variables to refine the metamodel’s predictive capabilities further.

Selecting an appropriate dataset is paramount to ensure our metamodel’s generalizability and relevance. In this study, we utilized five well-known heart datasets. These datasets have been extensively used in previous research, contributing valuable clinical information about heart disease and heart failure. Before combining these datasets, we thoroughly investigated their characteristics, ensuring compatibility in terms of the target variable (heart failure) and the set of standard features they shared. Combining disparate datasets introduces potential limitations and biases stemming from variations in data collection protocols, demographics, and healthcare practices across different geographical regions and time periods. We acknowledge that these inherent differences might influence the model’s generalization ability. To mitigate potential biases, we employed various strategies during the dataset integration process explained in the Data-Preprocessing section.

In addition to ensuring easy integration with medical environments, even those with limited resources, we employ machine learning baselines to maintain a lightweight model. However, deep learning demands more computational power, making it unsuitable for achieving a lightweight design.

Developing an accurate machine learning metamodel for heart failure prediction presents numerous challenges. The diverse range of risk factors, including underlying heart diseases, comorbidities, and lifestyle habits, necessitates the integration of heterogeneous data types and interactions. Feature selection becomes critical to extract relevant information while handling high-dimensional data and potential multicollinearity. Addressing data imbalance is crucial to avoid biased predictions, and generalization to unseen data requires external validation. Balancing predictive performance with interpretability is essential for clinical adoption, considering the model’s potential as a “black box”. Data quality and completeness are pivotal in ensuring reliable predictions, emphasizing the need for careful data preprocessing. Overcoming these challenges is vital to advance heart failure prediction and for fostering the model’s clinical utility in cardiology.

While the results of this research are promising, several limitations should be acknowledged. Future work for this paper includes conducting external validation of the developed metamodel using larger and more diverse datasets, evaluating its performance using additional metrics such as sensitivity, specificity, and AUC-ROC, exploring different feature selection techniques to enhance accuracy, developing interpretability techniques without compromising predictive accuracy, incorporating longitudinal data analysis to capture temporal patterns, integrating clinical notes, assessing the metamodel’s practical implementation in clinical settings, integrating external data sources for a comprehensive patient profile, and exploring the impact of the metamodel on patient care, outcomes, and healthcare systems. These avenues will further advance cardiac failure forecasting, improve patient care, and refine the metamodel’s performance and applicability in clinical practice.

## 6. Conclusions

This research presents a machine learning metamodel for cardiac failure forecasting based on clinical data. The metamodel demonstrates improved predictive accuracy and model robustness by integrating machine learning algorithms. Using a combined dataset from five well-known cardiac datasets enhances its generalizability and applicability. Evaluation results reveal that the metamodel outperforms other state-of-the-art models, with an accuracy of 87%. This development holds excellent potential for accurately identifying patients at risk of heart failure, enabling timely interventions and personalized treatment plans. Integrating machine learning techniques in clinical practice can significantly enhance patient care, improve outcomes, and reduce healthcare costs. Further studies can explore the integration of additional clinical variables and validate the metamodel using more extensive and diverse datasets to strengthen its reliability and generalizability. Overall, this machine learning metamodel significantly advances cardiac failure forecasting, potentially improving patient outcomes and saving lives through early detection and proactive management.

## Figures and Tables

**Table 1:** A Comparative Overview of Heart Disease Prediction Methods: Various methods and their performance on different datasets, including limitations.

Ref	Method	Dataset	Records Count	Outcome	Limitations
[19]	SVM, NN, Fuzzy Genetic, CART, and Random Forest	Custom dataset	136	CART was most effective in determining the type and degree of heart failure.	Small sample size prevented the proposed model from generalizing well. In determining severity, accuracy is fairly poor.
[20]	Modified Self Adaptive Bayesian algorithm (MSABA) and IoT	UCI	303	Used the pulse sensor of a smart watch to obtain data and find the disease.	Used only ECG data and blood pressure data for analysis and used fewer records for training.
[21]	Five active learning multi-label selection methods: random, MMC, adaptive, AUDI, and Quire	UCI	303	Accuracy in the generalisation of the learning model beyond the available data for the optimised label ranking model.	Fewer data were utilized for prediction and fine-tuning because the system is sophisticated.
[22]	Support Vector Machine (SVM)	UCI and Cleveland datasets	573	The performance of the proposed model was subsequently validated by comparing it to conventional models in 2022 using a number of performance criteria, and the componential load was cut in half.	The features used in the system were decreased from 14 to 6 to reduce the computational load, but sometimes the reduced features are also important for disease analysis.
[23]	Sine Cosine Weighted K-Nearest Neighbour (SCA_WKNN) algorithm	UCI	303	In comparison to WK-NN and K-NN, CA_WKNN obtains maximum accuracy of 4.59% and 15.61%, respectively. Blockchain-powered decentralised storage exceeds peer-to-peer storage in terms of maximum throughput by 25.03 percent.	The operational cost is contingent upon the number of transactions conducted within the system, making it expensive. As the data expands, it becomes necessary to limit the system’s learning capacity to a restricted data source to avoid incurring additional costs.
[24]	LR, ANN, SVM(support vector machine)	Korea National Health and Nutrition Examination Survey by smartwatches	6170	Out of the three models applied, the SVM gives the highest accuracy of 83.04% for six types of heart disease prediction.	As for the data collection using smartwatch sensors, all the necessary feature data are not available for proper heart disease prediction.

**Table 2:** Comparison of Model Performances with Reporting Features.

Model	Reporting Features	Precision (P) (%)	Recall (R) (%)	F1-Score (F) (%)
LGBM	accuracy	-	-	83
	macro avg	83	83	83
	weighted avg	83	83	83
Ridge	accuracy	-	-	86
	macro avg	86	86	86
	weighted avg	86	86	86
SVR	accuracy	-	-	70
	macro avg	70	70	70
	weighted avg	70	70	70
SGDC	accuracy	-	-	76
	macro avg	76	76	75
	weighted avg	77	76	75
KNN	accuracy	-	-	68
	macro avg	68	68	68
	weighted avg	68	68	68
GPC	accuracy	-	-	61
	macro avg	61	61	61
	weighted avg	61	61	61
Tree	accuracy	-	-	79
	macro avg	79	79	79
	weighted avg	79	79	79
Bagging	accuracy	-	-	86
	macro avg	87	86	86
	weighted avg	87	86	86
**Proposed model **	accuracy	-	-	87
	macro avg	87	87	87
	weighted avg	87	87	87

**Table 3:** The table illustrates the parameters and the number of parameters used in the datasets. Additionally, it shows the total data count for the entire dataset and presents the comparison of model results for different studies.

Study	Dataset	Parameters	Parameters Count	Records	Algorithm Used	Accuracy (%)
Simge et al. [8]		Age, Sex,			DT	60.9
		CP, trstbps, Thal,			Linear SVM	65.3
	UCI [12]	Chol, FBS, CA,	13	300	Quadritic SVM	65
		RestECG, thalach,			Cubic SVM	61.9
		Exang, OldPeak, Slope			Medium Gaussian SVM	67
					Ensamble Subpace Discriminant	67.7
Saba et al. [11]		Age, Sex,			DT	82.22
		CP, trstbps, Thal,			Linear Regression	82.56
	UCI [12]	Chol, FBS, CA,	13	300	Random Forest	84.17
		RestECG, thalach,			Naive bayes	84.24
		Exang, OldPeak, Slope			Logistic Regression (SVM)	84.85
Robert et al. [7]	UCI [12]	Age, CP, Sex, Trstbps, FBS, CA, Chol, Thalach, RestECG, Exang, Slope, Old Peak, Thal	13	300	Linear Regression	77
Ali et al. [9]		Age, Sex, CP, OldPeak, thalach,			Naive bayes	84
	UCI [12]	Chol, FBS, CA, RestECG,	13	300	KNN	80
		trstbps, Thal, Exang, Slope			SVM	83
Proposed System	fedesor-iano [25]	Age, chest pain type, sex, resting BP, fasting BS, cholesterol, resting ECG, maxHR, oldpeak, exercise angina, and ST-slope	11	918	metamodel	87

## Data Availability

The data present in this study are available on request from the author.
